# Evaluating the Effectiveness of a Commercial Portable Air Purifier in Homes with Wood Burning Stoves: A Preliminary Study

**DOI:** 10.1155/2011/324809

**Published:** 2011-01-27

**Authors:** Julie F. Hart, Tony J. Ward, Terry M. Spear, Richard J. Rossi, Nicholas N. Holland, Brodie G. Loushin

**Affiliations:** ^1^Department of Safety, Health, & Industrial Hygiene, Montana Tech of The University of Montana, 1300 West Park Street, Butte, MT 59701, USA; ^2^Center for Environmental Health Sciences, The University of Montana, Missoula, MT 59812, USA

## Abstract

Wood burning for residential heating is prevalent in the Rocky Mountain regions of the United States. Studies have shown that wood stoves can be a significant source of PM_2.5_ within homes. In this study, the effectiveness of an electrostatic filter portable air purifier was evaluated (1) in a home where a wood stove was the sole heat source and (2) in a home where a wood stove was used as a supplemental heat source. Particle count concentrations in six particle sizes and particle mass concentrations in two particle sizes were measured for ten 12-hour purifier on and ten purifier off trials in each home. Particle count concentrations were reduced by 61–85 percent. Similar reductions were observed in particle mass concentrations. These findings, although limited to one season, suggest that a portable air purifier may effectively reduce indoor particulate matter concentrations associated with wood combustion during home heating.

## 1. Introduction

In today's society, it is estimated that people may spend as much as ninety percent of time in indoor environments [1]. While numerous sources of ambient pollutants have been characterized, indoor pollutants, such as dust, smoke, pollen, and animal dander particulate matter, as well as various gaseous pollutants, have gained considerable attention in terms of potential adverse health effects. The United States Environmental Protection Agency (USEPA) considers indoor air pollution among the top five environmental health risks [2].

As the awareness of potential indoor air contaminants has increased, so have the marketing and sales of home air cleaning devices, with Americans spending 500 million dollars annually on whole house and portable air cleaners [3]. Whole house filtration systems are typically employed in the return duct of central heating, ventilating, and air conditioning (HVAC) systems or forced air heating systems. Portable room air cleaners or purifiers, as the name implies, are designed to be used in single rooms or specific areas within the home. These systems are an option when a heating system is not conducive to a whole house cleaner, such as the case with a wood burning stove or fireplace (which is typically located within a common area of the residence).

Portable air cleaners contain a fan that circulate room air and employ technologies such as mechanical filtration, electrostatic precipitation, ozone generation, and so forth. The efficiency of these various technologies is based on the relationship between the concentration of particles in the air entering the device and the concentration of particles in the air leaving the device. This is commonly referred to as single pass efficiency. While this method considers the efficiency associated with the filtering mechanism, air cleaner effectiveness (E) takes into consideration the volume of space in which the air cleaner is used [4]. 

 A minimum effectiveness value of 0.8 is recommended by the Association of Home Appliance Manufacturers [5] and is equivalent to an air cleaner capable of providing an equivalent volume of four to five clean air changes per hour [6]. Air cleaner effectiveness has been evaluated for various types of air cleaner technologies [4, 7–11]. These studies, which employed models or were conducted within test chambers with controlled aerosol generation, have demonstrated that variables such as particle size, air cleaner technology, air exchange rate, and position of the air cleaner in a room are factors influencing air cleaner effectiveness. 

The most common rating used by manufacturers for evaluating the performance of portable air cleaners is the Clean Air Delivery Rate (CADR) [5]. This rating is based on the measured decay rate of contaminant concentrations with the air cleaner operating compared with the measured decay rate of contaminant concentrations with the air cleaner off, multiplied by the volumetric airflow through the device. The particle removal rate effectiveness is evaluated for dust, tobacco smoke, and pollen (representing three particle size ranges) in a room size test chamber [5]. 

Wood burning for primary or supplemental home heating is prevalent in both rural and urban areas throughout the Northern Rocky Mountains. Wood smoke has been identified through source apportionment studies as a major source (>50%) of wintertime ambient PM_2.5_ in several rural valley locations in this region [12, 13]. Although wood stoves and fireplaces are vented to the outside, their use is associated with elevated pollutants in the indoor air, including particulate matter [14–16]. In addition to particulate matter generated indoors from wood burning, infiltration from outdoor environments may contribute substantially to indoor particulate matter concentrations [17, 18]. The application of portable air cleaners has been demonstrated to be effective in reducing indoor PM 2.5 concentrations associated with infiltration of wood smoke from residential wood burning, forest fire events, and prescribed burns [18, 19]. 

The acute and chronic health effects associated with woodsmoke from forest fire and residential wood burning are summarized in recent reviews by Naeher et al. [20] and Bølling et al. [21]. Epidemiology studies have revealed that young children are particularly susceptible to the effects of wood smoke with increased incidence of respiratory symptoms [22–24], asthma emergency department visits [25, 26], and asthma symptoms [27, 28]. Wood smoke has also been associated with increased cardiovascular emergency department visits [29]. The International Agency for Research on Cancer has concluded that indoor emissions from household combustion of biomass fuel (mainly wood) are probably carcinogenic to humans (Group 2A) [30]. Cellular studies have revealed proinflammatory responses to wood smoke quantified by cytokine release and cell number [31, 32]. In addition to inflammation, oxidative stress leading to lipid peroxidation and changes in blood coagulation factors have been observed [33].

While the nonclinical studies evaluating the effectiveness of air filtration have been positive, the clinical implications, with a primary focus on respiratory allergy/asthma symptoms, are unresolved [34]. An epidemiology study concluded that the use of high-efficiency particulate air (HEPA) portable air cleaners reduced the odds of reporting worsening respiratory symptoms during forest fire events [35]. A recent study revealed a significant improvement in microvascular function among a healthy elderly population associated with the use of a HEPA filtered portable air cleaner and subsequent PM 2.5 reductions [36].

The objective of this study was to evaluate the effectiveness of a relatively new portable air cleaning technology, electrostatic filters, in residential settings where wood burning was conducted as a primary or secondary source of space heating. Limited published literature is available regarding the effectiveness of air purifying systems in reducing indoor particulate concentrations associated directly with wood combustion in the home. Replacing older wood burning appliances with newer EPA-certified woodstove models has been shown in two studies [15, 37] to be an effective tool in reducing indoor PM_2.5_ from wood stoves, while in another study [16] this intervention did not result in a consistent reduction in indoor PM_2.5_. Aside from the mixed conclusions regarding the effectiveness of a woodstove change out in reducing indoor PM_2.5_, many households cannot afford this option. In addition, there is an abundance of inexpensive biomass fuel sources in the Northern Rocky Mountains when compared to the rising costs of fossil fuels. 

This study evaluated the viability of electrostatic filter room air cleaners as a relatively inexpensive intervention measure for reducing particulate matter concentrations associated with biomass burning during residential home heating. Information from this study will provide valuable information to consumers and public health officials regarding the effectiveness of this intervention measure in relation to the use of wood stoves. In addition, this intervention measure is currently being evaluated in a study involving asthmatic children living in homes where wood stoves are used as a heat source.

## 2. Methods

Research was conducted in spring of 2008, in two Butte, MT homes. Home A was a 125 m^2^ double-wide trailer that was constructed in 1976. The sole source of heat in this home was a 1979 Hearth Flo model wood burning stove. Home B was a 122 m^2^ conventional wood-frame stud constructed home that was built in 1967. The primary source of heat in Home B was a 2007 Lenox Elite forced air natural gas furnace fitted with a new 3M Filtrete pleated filter. The thermostat in Home B was set at a constant 10 degrees Celsius. A 1970s model Blaze King wood burning stove was used as a supplementary heat source in Home B. The Blaze King had been refurbished; therefore, the model and date of construction were unavailable. Both of the wood stoves, in Homes A and B, were not certified by the EPA for particulate emissions. One occupant resided in Home A, while two occupants resided in Home B.

Sampling was conducted in each home for ten 24 hour periods. In each of the 24 hour sampling periods, a FAP02-RS model 3M Filtrete portable air purifier fitted with an electrostatic filter was in operation with the instrument setting on high for twelve hours, or one half of the sample period duration. The remaining sample duration was conducted with the Filtrete air purifier turned off. The twelve-hour increment Filtrete on/off sample trials were randomly selected for each of the ten 24 hour sampling periods. 

**Figure 1:**
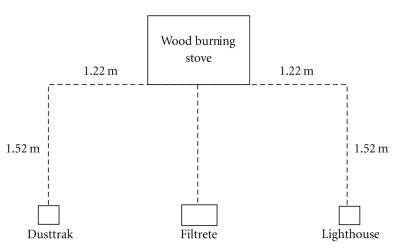
Configuration of the wood burning stove, Filtrete air purifier, DustTrak aerosol monitor, and Lighthouse particle counter in each home. An additional DustTrak was placed in a secondary location (bedroom) 5.84 meters from the wood stove in Home B.

The FAP02-RS model Filtrete is designed to operate in rooms up to 15.8 m^2^ (170 ft^2^), a condition met by the primary room sampled in each home. The published CADRs for this model are 3.6 m^3^/min (128 ft^3^/min), 2.9 m^3^/min (103 ft^3^/min), and 4.2 m^3^/min (149 ft^3^/min) for dust, tobacco smoke, and pollen, respectively [38]. Prior to the 10-day sample period in each home, a new 3M electrostatic filter was positioned in the air purifier. During each sampling event, the base of the air purifier was positioned 0.86 m off the floor 1.5 m away from the wood burning stove. A Lighthouse model 3016 direct reading laser particle counter and a TSI DustTrak model 8520 aerosol monitor were also positioned 1.95 m away from the wood burning stove (Figure 1). The base of these two instruments was placed 1.2 meters from the floor. In addition to the sampling configuration described above, an additional DustTrak was placed in a secondary location (bedroom) 0.86 m from the floor and 5.84 m from the wood stove in home B for the 10-day sample period. 

Both the Lighthouse particle counter and the DustTrak were factory calibrated prior to the study. The flow rate of both instruments was 2.83 L/m. Manufacturer instructions were followed for cleaning and calibrating the instruments prior to use. Both instruments were programmed to report data in five-minute intervals. During five sampling periods, the DustTrak was fitted with a 1.0 micron (*μ*m) inlet and during the remaining five sampling periods, a 2.5 *μ*m inlet was employed. The Lighthouse particle counter measures particle counts at six simultaneous cutpoints: 0.3, 0.5, 1, 2.5, 5, and 10 *μ*m.

The wood burned in each home was locally harvested lodgepole pine (*Pinus contorta* Dougl.). The mass of the wood burned was recorded for each trial with a Health O Meter model HDM560DQ-05 X209BN scale. The amount of wood burned was determined by the desired thermal comfort of the occupants. Ambient temperatures for each sample period were recorded from the National Weather Service. Indoor temperatures and relative humidity for each sample period were measured with the Lighthouse. 

Indoor activities that may influence measured PM concentrations were documented on a daily log sheet. These activities include lighting the stove, adding wood, cleaning the stove, cooking food, and cleaning tasks. All home occupants were nonsmokers.

### 2.1. Data Analysis

Mean particle/m^3^ concentrations, *μ*g/m^3^ concentrations, and upper and lower confidence intervals are presented for 12-hour air purifier on and off trials. For comparison, data were log-transformed to approximate normality and multiple regression tests were conducted (Minitab Version 15, USA). The effects of air purifier on/off, day/night, week, mass of wood combusted, relative humidity, and temperature were evaluated.

## 3. Results and Discussion

Throughout the sampling program, twenty 12-hour trials were conducted in each home, with a mean of 144 data points per 12-hour trial. Ten trials were collected with the air purifier operating “on”, and 10 trials were collected with the air purifier off in each home. The air purifier on/off schedule in relation to day versus night sampling was randomly selected. In each home, 5 air purifier “on” trials were conducted during the night (8:00 PM to 8:00 AM), and 5 air purifier on trials were conducted during the day (8:00 AM to 8:00 PM). 

The mean mass of wood combusted in Home A per 12-hour trial was 11.9 kg (SD = 7.2), while the mean ambient temperature, mean indoor temperature, and relative humidity were 1.8°C (SD = 4.33), 23.9°C (SD = 1.7), and 18.4% (SD = 1.9), respectively. The occupant of Home A remained in the home for the majority of the sample trial durations and added wood to the stove at a mean rate of once every two to three hours during daytime conditions. During the night, wood was typically added near midnight and the fire was restarted or wood was added again during the early AM hours. Activities in the home during the sample periods included very limited cooking (one day where grilling occurred) and no sweeping or vacuuming. 

In Home B, a mean mass of 5.81 kg (SD = 1.66) of wood was burned per 12-hour sample trial duration. This mass of wood burned was significantly lower (*P* = .020) than the mass of wood burned in Home A and may be related to the fact that Home B occupants relied on the wood stove as a secondary source of heat and/or that the mean ambient temperature of 5.4°C (SD = 3.3) recorded during sampling in Home B was significantly higher (*P* = .032) than the mean ambient temperature associated with sampling in home A. The mean indoor temperature and relative humidity recorded in Home B were similar to those recorded in Home A at 20.29°C (SD = 1.6) and 26.81% (SD = 1.2), respectively. Home B occupants both worked outside the home and used the supplemental wood stove in the AM (8:00–10:00 AM) and evening (6:30–11:00 PM) hours only. Activities in home B during the sample periods included limited cooking (two events where grilling and baking both occurred) and cleaning (four events where sweeping occurred).

**Figure 2:**
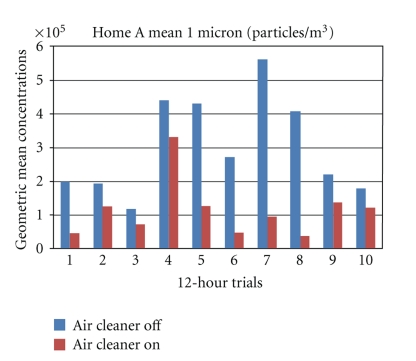
Geometric mean particle count concentrations (particle/m^3^) observed with the portable air purifier off and on at the one *µ*m cutpoint for each 12-hour trial conducted in home A. Concentrations recorded with the air purifier on were consistently lower than concentrations recorded with the air purifier off. Similar observations were made in terms of particle concentration reductions with the air cleaner on at the other cutpoints.

Mean 12-hour particle count and particle mass concentrations were consistently lower when the portable air purifier was on verses when the air purifier was off. An example of this trend is presented in Figure 2. Mean particle count concentration (particles/m^3^) results obtained with the air purifier off and on for the 20 sampling trials conducted in Home A are presented in Table 1. These data, along with lower and upper confidence intervals and percent reductions in particle concentrations are presented for six particle size cutpoints. The effectiveness of the air cleaner was demonstrated at all particle cutpoints, ranging from 61% to 66% reduction in particle count concentrations. Significant reductions were observed even in the lowest particle size range (0.3 to 0.5 *μ*m), a range that has been demonstrated to be the least likely to be filtered [8]. Significant differences (*P* < .05) were observed between day and night particle count concentrations in the 2.5 to 10.0 *μ*m cutpoints; therefore, these mean concentrations are presented independently (Table 1, rows 4–8).

Mean particle count concentration (particles/m^3^) results obtained with the air purifier off and on for the 20 sampling trials conducted in Home B are presented in Table 2. The effectiveness of the air cleaner in Home B ranged from 78% to 85% reduction in particle count concentrations. Significant differences (*P* < .05) between day and night particle count concentrations were observed at the 10.0 *μ*m cutpoints; therefore, these mean concentrations are presented independently (Table 2, rows 6 and 7).

In addition to a Lighthouse particle counter, a TSI DustTrak was used in both homes to measure particle mass concentrations. Mean particle mass concentrations, lower and upper confidence intervals, and mass concentration percent reductions are illustrated for both homes in Table 3. In Home A, the DustTrak was placed near the Lighthouse as illustrated in Figure 1. During the first five sampling periods in Home A, the DustTrak was fitted with a 1 *μ*m inlet, while during the second five sampling periods, a 2.5 *μ*m inlet was used. Reductions in particle mass concentrations at these cutpoints were similar to the effectiveness revealed via particle count concentrations, ranging from 58–76% reduction in the 1.0 and 2.5 *μ*m cutpoints, respectively (Table 3, rows 1 and 2). A strong, positive correlation (*r* = 0.74) was observed between particle count and particle mass concentration data at both cutpoints (not shown in Table 3). 

Two TSI DustTraks were also used along with the Lighthouse particle counter in Home B. One DustTrak was positioned in the location illustrated in Figure 1, and the other was positioned in a bedroom approximately six meters from the wood stove. As with Home A, during the first five sampling periods, a 1.0 *μ*m inlet was used on both DustTraks in Home B, and during the next five sampling periods, a 2.5 *μ*m inlet was used. The DustTrak positioned near the woodstove revealed a 76% reduction in particle mass concentrations between the air purifier on and air purifier off trials at the 2.5 *μ*m cutpoint. The effectiveness revealed at this cutpoint is slightly lower than the reductions observed at the same cutpoint with the Lighthouse. At the 1.0 *μ*m cutpoint, the air purifier appeared to reduce particle mass concentrations (55%); however, a significant difference between air purifier on and air purifier off concentrations was not observed. This was attributed to the high variability in the data at this cutpoint and may be associated with the limited sample size. It is interesting to note that the secondary DustTrak, positioned in a separate room, revealed particle mass concentration reductions of 74 and 75%, respectively. A strong, positive correlation (*r* = 0.95) was observed at the 1.0 *μ*m cutpoint between particle count (measured near wood stove) and particle mass concentration data obtained with the bedroom DustTrak (not shown in Table 3). At the 2.5 *μ*m cutpoint, the correlation observed between the particle count and particle mass concentration data measured near the wood stove was (*r* = 0.90), while the correlation observed at this same particle size between the particle count concentration measured near the wood stove and particle mass concentration measured in the bedroom was similar (*r* = 0.88).

The particle count and particle mass concentration reductions observed in both homes at the 2.5 *μ*m cutpoint (61–85% reduction in particle count concentration and 76–76% reduction in particle mass concentration) were similar to the results observed in the Henderson et al. [19] and Barn et al. [18] smoke infiltration studies. Indoor PM_2.5_ particle mass concentrations were reduced by 63–88% with the use of multiple electrostatic precipitating portable air cleaners in operation during wildfire and prescribed burn events [19], illustrating that portable air cleaner operation may be an effective method for reducing indoor PM_2.5_ associated with forest fires. Barn et al. [18] assessed air cleaner effectiveness by measuring decreased PM_2.5_ infiltration associated with air cleaner use. Approximately, 30 percent of outdoor PM_2.5_ was infiltrated indoors during the winter residential wood burning season. The use of a HEPA filtered portable air cleaner reduced wintertime PM_2.5_ infiltration by 65% (±35%).

## 4. Conclusion

This study suggests that a portable air cleaner may be a viable option for reducing particle concentrations in homes with wood stoves utilized for primary and secondary space heating requirements. Reductions in particle count concentrations in Home A, where a wood stove was the sole space heating source, ranged from 61–66%, while particle mass concentrations in this home were similar with 58 and 76% reductions in the 1 and 2.5 *μ*m particle sizes, respectively.

Although Home B utilized a wood stove as a supplemental space heating source in the early morning and evening hours only, baseline (air cleaner off) particle count concentrations were consistently higher at all six particle size ranges than baseline concentrations in Home A. Reductions in particle count concentrations were also greater in Home B (78–85%) than Home A. Mean particle mass concentrations and percent reduction in the two homes were similar.

While the aim of this study was to evaluate the effectiveness of a portable air purifier in reducing particle concentrations associated with wood combustion, the particles measured in both homes were obviously derived from numerous sources, including wood stoves. The particle concentration reductions observed in both homes at the ≤2.5 *μ*m cutpoint are indicative of the air cleaner effectiveness for wood smoke derived particles.

In both homes, there was a strong, positive correlation between particle count concentrations and particle mass concentrations, revealing similar results between two separate monitoring techniques in evaluating the air cleaner effectiveness. In addition to the strong correlation observed in these data between two instruments positioned in the same room as the wood stove, in Home B, this same trend was observed between bedroom particle mass concentration data and particle count concentration data collected near the wood stove. This suggests that a portable air purifier may effectively reduce particle concentrations in secondary rooms.

This study has several limitations. The effectiveness of a portable air purifier was evaluated in only two homes. The study was conducted during ten early spring days in each home, negating potential seasonal differences in particle concentrations. Source apportionment of particles was not conducted, and the infiltration rate into homes was not quantified; therefore, the source of particles was not defined. Further studies are needed to further define the effectiveness of portable air cleaners in residential settings.

In conclusion, a portable air cleaner did reduce particle count and particle mass concentrations of several particle sizes in homes that utilized wood stoves for primary or secondary space heating requirements. This suggests that a portable air cleaner may be a relatively inexpensive, effective mitigation measure to reduce particle concentrations and the risk of associated health effects in homes that rely on wood burning for space heating.

## Figures and Tables

**Table 1:** Home A mean concentrations (particle/m^3^) measured with the Lighthouse, lower and upper confidence intervals, and percent changes (%).

	Air purifier off			Air purifier on				
Particle cutpoint (*μ*m)	Mean concentration (p/m^3^)	LCI (p/m^3^)	UCI (p/m^3^)	Mean concentration (p/m^3^)	LCI (p/m^3^)	UCI (p/m^3^)	Percent change	*P* value
0.3	21,921,972	15,294,441	31,421,408	7,579,820	5,288,261	10,864,379	−65%	.000
0.5	2,006,696	1,317,175	3,054,114	684,881	477,825	2,006,696	−66%	.001
1	257,043	169,058	390,819	96,858	63,768	147,267	−63%	.003
2.5								
Day	67,643	43,783	104,402	26,716	17,309	41,274	−61%	.002
Night	30,577	19,791	47,193	12,076	7,824	18,657	−61%	.002
5								
Day	14,827	10,451	21,034	5,530	3,898	7,846	−63%	.000
Night	4,565	3,218	6,476	1,703	1,203	2,416	−63%	.000
10								
Day	2,502	1,895	3,616	973	673	1,405	−61%	.000
Night	652	452	942	253	176	366	−61%	.000

**Table 2:** Home B mean concentrations (particle/m^3^) measured with the Lighthouse, lower and upper confidence intervals, and percent changes (%).

	Air purifier off			Air purifier on				
Particle cutpoint (*μ*m)	Mean concentration (p/m^3^)	LCI (p/m^3^)	UCI (p/m^3^)	Mean concentration (p/m^3^)	LCI (p/m^3^)	UCI (p/m^3^)	Percent change	*P* value
0.3	26,010,218	16,452,697	41,119,787	4,542,554	2,870,509	7,181,365	−82%	.001
0.5	2,533,976	1,938,451	3,312,786	441,088	337,425	576,655	−83%	.000
1	569,264	423,285	765,588	85,974	63,927	115,636	−85%	.000
2.5	206,282	126,121	337,729	32,663	19,950	53,477	−84%	.000
5	31,351	16,933	58,047	6,039	3,262	11,181	−81%	.002
10								
Day	7,347	4,209	12,823	1,621	857	3,072	−78%	.002
Night	1,239	654	2,347	273	157	478	−78%	.002

**Table 3:** Home A and B particle mass concentrations (*μ*g/m^3^) measured with the DustTrak, lower and upper confidence intervals, and percent changes (%).

		Air purifier off			Air purifier on				
Home	Particle cutpoint (*μ*m)	Mean concentration (*μ*g/m^3^)	LCI (*μ*g/m^3^)	UCI (*μ*g/m^3^)	Mean concentration (*μ*g/m^3^)	LCI (*μ*g/m^3^)	UCI (*μ*g/m^3^)	Percent change	*P* value
A	1.0	8.85	3.94	13.8	3.72	1.15	6.28	−58%	.040
	2.5	13.98	5.93	22.03	3.30	1.09	5.51	76%	.025
B	1.0	7.08	0.83	13.32	3.21	0	7.60	−55%	.238
	2.5	13.60	8.10	19.09	3.26	2.23	4.29	−76%	.007
B	1.0	6.86	2.95	10.77	1.83	0.99	2.61	−74%	.025
Secondary*	2.5	8.88	5.27	12.49	2.22	1.63	2.82	−75%	.007

*Secondary location (bedroom) in Home B 5.84 meters from wood stove.

## References

[1] Alford G (2005). The buzz on room air cleaners: how effective are they for management of allergies and asthma?. *Asthma Magazine*.

[2] United States Environmental Protection Agency Guide to Air Cleaners. http://www.epa.gov/iaq//pubs/airclean.html.

[3] Consumer Reports http://www.consumerreports.org/cro/appliances/heating-cooling-and-air/air-purifiers/air-purifier-buying-advice/air-purifier-getting-started/air-purifier-getting-started.htm.

[4] Miller-Leiden S, Lobascio C, Nazaroff WW, Macher JM (1996). Effectiveness of in-room air filtration and dilution ventilation for tuberculosis infection control. *Journal of the Air and Waste Management Association*.

[5] Association of Home Appliance Manufacturers (AHAM) Method for Measuring Performance of Portable Household Electric Room Air Cleaners.

[6] Shaughnessy RJ, Sextro RG (2006). What is an effective portable air cleaning device? A review. *Journal of Occupational and Environmental Hygiene*.

[7] Cheng YS, Lu JC, Chen TR (1998). Efficiency of a portable indoor air cleaner in removing pollens and fungal spores. *Aerosol Science and Technology*.

[8] Offermann FJ, Sextro RG, Fisk WJ (1985). Control of respirable particles in indoor air with portable air cleaners. *Atmospheric Environment*.

[9] Ward M, Siegel JA, Corsi RL (2005). The effectiveness of stand alone air cleaners for shelter-in-place. *Indoor Air*.

[10] Waring MS, Siegel JA, Corsi RL (2008). Untrafine particulate removal and generation by portable air cleaners. *Atmospheric Environment*.

[11] Novoselac A, Siegel JA (2009). Impact of placement of portable air cleaning devices in multizone residential environments. *Building and Environment*.

[12] Ward TJ, Rinehart LR, Lange T (2006). The 2003/2004 Libby, Montana PM2.5 source apportionment research study. *Aerosol Science and Technology*.

[13] Ward T, Lange T (2010). The impact of wood smoke on ambient PM in northern Rocky Mountain valley communities. *Environmental Pollution*.

[14] Robin LF, Lees PSJ, Winget M (1996). Wood-burning stoves and lower respiratory illnesses in Navajo children. *Pediatric Infectious Disease Journal*.

[15] Ward T, Palmer C, Bergauff M, Hooper K, Noonan C (2008). Results of a residential indoor PM2.5 sampling program before and after a woodstove changeout. *Indoor Air*.

[16] Allen RW, Leckie S, Millar G, Brauer M (2009). The impact of wood stove technology upgrades on indoor residential air quality. *Atmospheric Environment*.

[17] Larson T, Gould T, Simpson C, Liu LJS, Claiborn C, Lewtas J (2004). Source apportionment of indoor, outdoor, and personal PM in Seattle, Washington, using positive matrix factorization. *Journal of the Air and Waste Management Association*.

[18] Barn P, Larson T, Noullett M, Kennedy S, Copes R, Brauer M (2008). Infiltration of forest fire and residential wood smoke: an evaluation of air cleaner effectiveness. *Journal of Exposure Science and Environmental Epidemiology*.

[19] Henderson DE, Milford JB, Miller SL (2005). Prescribed burns and wildfires in Colorado: impacts of mitigation measures on indoor air particulate matter. *Journal of the Air and Waste Management Association*.

[20] Naeher LP, Brauer M, Lipsett M (2007). Woodsmoke health effects: a review. *Inhalation Toxicology*.

[21] Bølling AK, Pagels J, Yttri KE (2009). Health effects of residential wood smoke particles: the importance of combustion conditions and physiochemical particle properties. *Particle and Fibre Toxicology*.

[22] Honicky RE, Osborne JS, Akpom CA (1985). Symptoms of respiratory illness in young children and the use of wood-burning stoves for indoor heating. *Pediatrics*.

[23] Morris K, Morganlander M, Coulehan JL, Gahagen S, Arena VC (1990). Wood-burning stoves and lower respiratory tract infection in American Indian children. *American Journal of Diseases of Children*.

[24] Triche EW, Belanger K, Beckett W (2002). Infant respiratory symptoms associated with indoor heating sources. *American Journal of Respiratory and Critical Care Medicine*.

[25] Schwartz J, Slater D, Larson TV, Pierson WE, Koenig JQ (1993). Particulate air pollution and hospital emergency room visits for asthma in Seattle. *American Review of Respiratory Disease*.

[26] Norris G, YoungPong SN, Koenig JQ, Larson TV, Sheppard L, Stout JW (1999). An association between fine particles and asthma emergency department visits for children in Seattle. *Environmental Health Perspectives*.

[27] Koenig JQ, Larson TV, Hanley QS (1993). Pulmonary function changes in children associated with fine particulate matter. *Environmental Research*.

[28] Yu O, Sheppard L, Lumley T, Koenig JQ, Shapiro GG (2000). Effects of ambient air pollution on symptoms of asthma in seattle-area children enrolled in the CAMP study. *Environmental Health Perspectives*.

[29] Sarnat JA, Marmur A, Klein M (2008). Fine particle sources and cardiorespiratory morbidity: an application of chemical mass balance and factor analytical source-apportionment methods. *Environmental Health Perspectives*.

[30] Straif K, Baan R, Grosse Y, Secretan B, El Ghissassi F, Cogliano V (2006). Carcinogenicity of household solid fuel combustion and of high-temperature frying. *The Lancet Oncology*.

[31] Karlsson HL, Ljungman AG, Lindbom J, Möller L (2006). Comparison of genotoxic and inflammatory effects of particles generated by wood combustion, a road simulator and collected from street and subway. *Toxicology Letters*.

[32] Kocbach A, Herseth JI, Låg M, Refsnes M, Schwarze PE (2008). Particles from wood smoke and traffic induce differential pro-inflammatory response patterns in co-cultures. *Toxicology and Applied Pharmacology*.

[33] Barregard L, Sällsten G, Gustafson P (2006). Experimental exposure to wood-smoke particles in healthy humans: effects on markers of inflammation, coagulation, and lipid peroxidation. *Inhalation Toxicology*.

[34] Sublett JL, Seltzer J, Burkhead R, Williams PB, Wedner HJ, Phipatanakul W (2010). Air filters and air cleaners: rostrum by the American Academy of Allergy, Asthma & Immunology Indoor Allergen Committee. *Journal of Allergy and Clinical Immunology*.

[35] Mott JA, Meyer P, Mannino D (2002). Wildland forest fire smoke: health effects and intervention evaluation, Hoopa, California, 1999. *Western Journal of Medicine*.

[36] Bräuner EV, Forchhammer L, Møller P (2008). Indoor particles affect vascular function in the aged: an air filtration-based intervention study. *American Journal of Respiratory and Critical Care Medicine*.

[37] Bergauff MA, Ward TJ, Noonan CW, Palmer CP (2009). The effect of a woodstove changeout on ambient levels of PM and chemical tracers for woodsmoke in Libby, Montana. *Atmospheric Environment*.

[38] Association of Home Appliance Manufacturers (AHAM) http://www.ahamdir.com/dirsvc/aham.nsf/fraAirCln?OpenFrameset&pgm=Room%20Air%20Cleaners.

